# Is left-behind a real reason for children’s social cognition deficit? An fNIRS study on the effect of social interaction on left-behind preschooler’s prefrontal activation

**DOI:** 10.1371/journal.pone.0254010

**Published:** 2021-09-17

**Authors:** Keya Ding, Chuanjiang Li, Huibin Jia, Mingming Zhang, Dongchuan Yu

**Affiliations:** 1 Key Laboratory of Child Development and Learning Science of Ministry of Education, School of Biological Science and Medical Engineering, Southeast University, Nanjing, China; 2 Division of Child and Adolescent Psychiatry, Washington University in St. Louis, St. Louis, MO, United States of America; 3 Hangzhou College of Early Childhood Teacher’s Education, Zhejiang Normal University, Hangzhou, China; 4 School of Psychology, Henan University, Kaifeng, China; 5 School of Psychology, Shanghai Normal University, Shanghai, China; 6 The Third Affiliated Hospital of Zhengzhou University, Zhengzhou, Henan, China; University of Iowa, UNITED STATES

## Abstract

The left-behind phenomenon, caused by parent out-migration, has become a common social issue and might lead to long-term and potential risks for children in rural areas of China. It is important to investigate the effect of social interaction on prefrontal activation of left-behind children in China because of possible effects of parent out-migration on children’s social cognition. We recruited 81 rural Chinese preschoolers aged 52–76 months (mean = 64.98 ± 6.321 months) preschoolers with three different statuses of parental out-migration (including non-, partially, and completely left-behind children). Using functional Near-Infrared Spectroscopy (fNIRS), we compared behavior and brain activation and in three groups (non-, partially-, completely-left-behind children) under two different social interaction conditions (child-teacher and child-stranger situation). Results revealed that initiating joint attention (IJA) may evoke higher brain activation than responding to joint attention (RJA) in the prefrontal cortex (PFC), especially in the case of initiating joint attention with the stranger. In addition, the activation of joint attention was positively correlated with children’s language score, cognitive flexibility, and facial expression recognition. More importantly, partially-left-behind children evoked higher brain activation in the IJA condition and presented a higher language level than completely/non-left-behind children. The current study provides insight into the neural basis of left-behind children’s development and revealed for the first time that family economic level and left-behind status may contribute to the lower social cognition.

## Introduction

Parents’ migration to seek work opportunities is a main cause of parent-child separations. Parental care affects the behavior of children [1] and even the maturation of the neural system that supports children’s psychological and emotional development. Studies in Romanian orphanages indicate that early global deprivation of institutionalized children might result in persistent mild neurocognitive impairment, and attention and social deficits [2, 3]. In the rural area of China, millions of laborers migrate to cities for better job opportunities [4]. Due to long working hours, insufficient economic ability, and the limitation of the urban and rural household registration system, they have to leave their children behind in the rural home, which leads to the emergence of a special group of left-behind children in the process of large-scale population mobility in China. By 2018, there were 6.97 million left-behind children in rural areas, of which the largest number of left-behind children in rural areas are between the ages of 6 and 13, more than 50% of all other age groups. Moreover, 96 percent of left-behind children are taken care of by their grandparents, and 4 percent are in the care of other relatives and friends [5]. In China, a child is considered as ‘left-behind’, if he/she is between 0 to 17 years old and lives without one or both of their parents for more than three consecutive months due to parent(s) who have been working away from home, and under the sole supervision of other relatives [4, 5]. In our research, we classified children into three categories, completely-left-behind children (meaning that both parents leave for cities and the children are left behind to live with a grandparent, relative, or family friend), partially-left-behind children (who stay with one parent in the rural village while another parent-often the father-leaves for work in the urban area), and non-left-behind children (who remain in the rural village with both parents) [6, 7].

Previous research in left-behind children presented a series of deficits in social cognitive development. For example, a study of 3–6 year-old children, left-behind children showed a poorer interaction with caregivers than non-left-behind children, and demonstrated lower social skill, vocabulary, and executive function levels compared to rural children [8]. Another study identified that being left behind may be a significant predictor of interaction status, victimization, and emotional distress [9]. Nevertheless, 43.6% of left-behind children aged 4–7 years from rural areas reported emotional or behavioral problems [10]. Thus far, most studies have only used behavioral tools to assess children. There are no imaging studies focus on brain activity underlying the cognitive development of left-behind children in China. Therefore, it is of interest to investigate biological indicators to help understand the development of left-behind children.

Joint attention plays a critical role in social interaction, and it has been termed a ‘window into social cognition’ [11]. Joint attention is the ability to start with basic perceptual and sensorimotor functions and develops toward the complex integration of other cognitive processes. Joint attention is imperative in advanced adult functioning and plays a crucial role in children’s language development and general learning. Joint attention can be divided into two types, i.e., Initiating Joint Attention (IJA) and Responding to Joint Attention (RJA). The two different types of joint attention indicate the unique process of social cognition and social learning [12]. Moreover, prior researches suggest that executive function and social-emotional ability may be associated with joint attention [13, 14]. Schilbach et al. (2010) and Pfeiffer et al. (2014) observed that self-initiated joint attention may involve areas associated with motivation and emotion processing and IJA has evoked more neural activity than RJA in adults [15, 16]. Other studies also demonstrated the difference of brain responses in IJA and RJA conditions [17, 18], which reported increased IJA-related activation of the right Inferior Frontal Gyrus (IFG) and Dorsolateral Prefrontal Cortex (DLPFC) being associated with cognitive flexibility [19]. However, despite well-designed longitudinal studies of infant and brain function studies in adults, very little research has been conducted to examine neural correlates of joint attention in preschool children, especially in left-behind groups. Consequently, due to its safety, portability, relatively high temporal resolution, and insensitivity to head movement, we recruited fNIRS for the joint attention task in the current study to evaluate the effect of joint attention on prefrontal activation in left-behind children [20].

Social behavior is highly dependent on which people interact and their relationships with each other. Studies with children, adolescents, and adults revealed that brain activation of social processing networks is enhanced under the familiar condition as compared to unfamiliar conditions [21, 22]. It is conceivable that familiar interaction partners may increase the motivational engagement in social interactions, since joint attention may employ modulating the associated neural systems. Taken together, prior research has provided evidence that parent-child separation may affect children’s development. However, whether and how the lack of direct parental care in childhood affects social cognition at the neural level of left-behind preschoolers remains unclear. In addition, previous studies on joint attention have not addressed the important aspect of reciprocal interaction in left-behind preschoolers. Thus, the present study was designed to (1) investigate the activation differences of joint attention type (i.e., IJA and RJA) and familiarity of the interaction partner (teacher and stranger); (2) evaluate the activation differences among three groups of preschoolers (completely-left-behind children, partially-left-behind children, and non-left-behind children) on joint attention tasks and other cognitive tasks (i.e., language, cognitive flexibility and facial expression recognition); and (3) assess the correlations between joint attention and other social cognitive abilities.

## Methods

### Procedure

All study procedures were approved by the Institutional Review Board at Southeast University. Online informed consent was obtained from all caregivers and oral consent was obtained from all children. All participants were recruited from 6 classrooms of a private kindergarten in a rural village of Guangxi province, China. The interested family contacted the teacher to understand the research content and completed a brief screen to determine eligibility. Children were excluded from participants in this study if they had a history of a neurological disorder, loss of consciousness, or sensory impairments, autism spectrum disorder, or an intellectual disability when recruited. Left-behind children were recruited if their parents/parent had been gone for over 3 months according to the definition of left-behind children. Each child received an age-appropriate toy after completing the study.

### Participants

A total of 89 children (51 boys) consented to participate in the study. 81 participants (44 boys) including 26 completely-left-behind children, 19 partially-left-behind children, and 36 non-left-behind children between the ages of 52–76 months (mean = 64.98 ± 6.321 months) had both fNIRS and behavioral usable data. Loss of fNIRS data was due to poor contact of the sensors with the scalp (N = 3), and /or too much movement during data collection (N = 4), or participants refused to attend the task (N = 1).

### Questionnaire

Teachers were asked to report the parent-child separation information of the participants. The Family Basic Information Questionnaire (online questionnaire) was conducted to collect family monthly income. The income was coded by the following rule: 1) a score of 1 represents a monthly income RMB 2k or below, 2) a score of 2 an income of RMB 2k-4k, 3) a score of 3 an income of RMB 4k-6k, 4) a score of 4 an income of RMB 6-8k, and 5) a score of 5 an income of RMB 8k or over.

### Behavioral tasks

The intelligence and Chinese receptive language of preschoolers were evaluated before doing tasks measured by Combined Raven’s Test (CRT) [23] and Peabody Picture Vocabulary Test-Revised (PPVT) respectively [24]. Then, two tablet-based games (TBG), Small or Big Pear Game (SBPG), and Facial Expression Recognition Task (FERT), were conducted to evaluate cognitive flexibility and social-emotional ability respectively.

1. Small or Big Pear game: Children were required to press the left basket (key) as fast as possible after a big pear presented in stage one; press the right basket as fast as possible after a small pear presented in stage two; big or small pear will randomly present in stage three, children were asked press the left or right basket according to the rule of the first two stages: small pear for the right basket, and big pear for the left basket (3 stages, 15 trials in each stage). This task was designed based on traditional Stroop [25] and modified to be engaging for the preschool age group to assess cognitive flexibility.
2. Facial expression recognition task: Children were required to point out the correct facial expression according to audio instructions (10 trials). All pictures of the emotional faces were select from the Mind Reading database [26].

### Joint attention task

**Fig 1:**
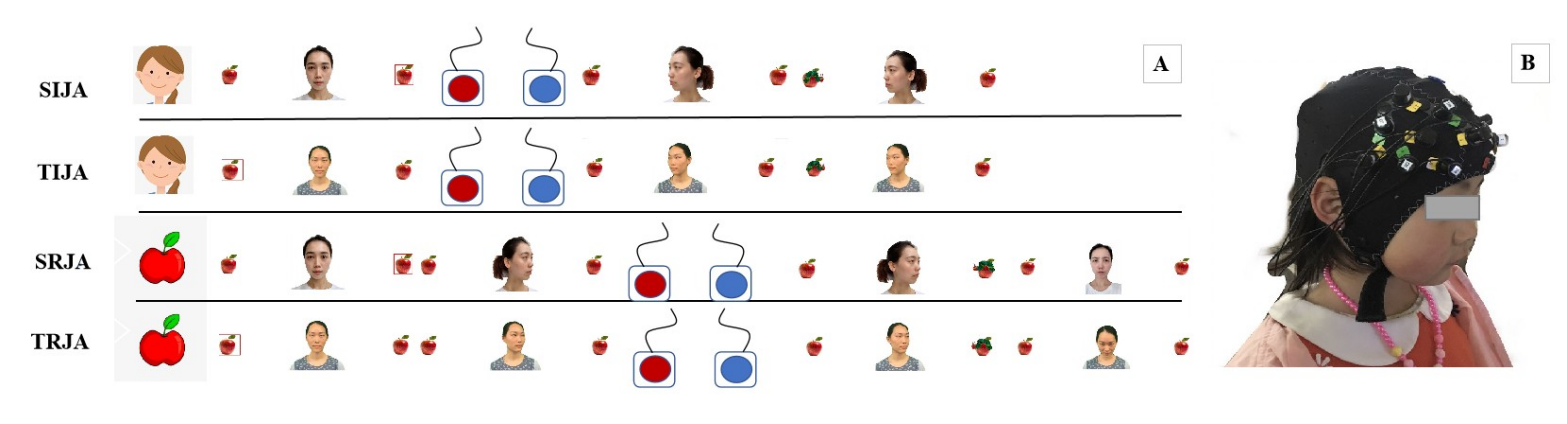
Joint attention task schematic. (A) The procedure of the joint attention task includes four experimental conditions (i.e., SIJA, TIJA, SRJA, and TRJA) (B) An example of a child with an fNIRS cap on (See S1 Fig in S1 File for consent form). SIJA = stranger-initiating joint attention; TIJA = teacher-initiating joint attention; SRJA = stranger-responding to joint attention; TRJA = teacher-responding to joint attention.

Based on previous joint attention tasks [15, 27], in combination with the aims of the current study, we designed a game-like, child-friendly paradigm to explore preschooler’s brain activation in IJA and RJA conditions under two interactional conditions (unfamiliar and familiar interacting partners). Considering the particularity of left-behind children, whose teacher played an important role in their daily learning, we took the teacher as a familiar interacting partner in the current research. The picture stimulus of the interaction partner was either a woman stranger (identical for all participants) or preschoolers’ key teachers. Key teachers from 6 classrooms (1 key teacher for each classroom) were photographed and implemented in the paradigm in the following way: before the scanning session, we took five pictures of teachers with gaze shifts towards the required positions and emotions (i.e., straight, left, right, two feedback photos). All preschoolers completed 2*2 (stranger/teacher*IJA/RJA) conditions, i.e., stranger IJA condition, stranger RJA condition, teacher IJA condition, and teacher RJA condition. All conditions were guided by a cue (pictures of a person or an apple) on the screen signal initiating or responding to a person (stranger/teacher). Preschoolers were familiarized with the instructions and practice before the formal start to ensure that they understood the task. The procedure is as shown in Fig 1., in which all conditions of the task are repeated three times with a total of 12 blocks (including 6 trials each block). The task lasts seven to eight minutes and was presented on a 14-inch computer screen (resolution of 1024*768) with a white background.

The joint attention task was conducted with E-prime (Psychology Software Tools Inc., Pittsburgh, PA, USA, Version 1.0) stimulation presentation software to demonstrate the experimental protocol in a block design during functional Near-Infrared Spectroscopy (fNIRS) imaging. In the RJA trial, a prompt cartoon person was present in the midline of the screen for 500ms. The child was instructed to follow the interacting partner’s gaze (600ms) towards to left or right via press the “left” or “right” button (maximum of 2000ms) in front of them as quickly and accurately as possible. The buttons with red and blue to distinguish, connect to the laptop via two USB ports. At the end of each interaction, 500ms feedback was present between each trial corresponding to the child’s correct and incorrect response. Indeed, the feedback correct response was the teacher/ stranger’s happy face while the incorrect feedback was the teacher/ stranger’s sad face. After that, a red cross (fixation) was present for 500ms before the next trial. In the IJA trial, a prompt apple picture stimulus was present in the middle of the screen for 500ms, and then a teacher/stranger’s picture with two apples in the left and right stimulus was present in the midline. Compare to the RJA trial, the stimulus of the teacher/stranger was looking forward, neither left nor right. The child could freely choose to shift the target person’s gaze by press the “left” or “right” button (maximum of 2000ms). The target person would gaze left or right according to the choice of the child, meanwhile, provided verbal feedback “Yeah!” to indicate the teacher/stranger followed the child’s gazing. The formal experiment began when the child’s accuracy exceeded 80% in the practice phase.

### fNIRS acquisition and processing

**Fig 2:**
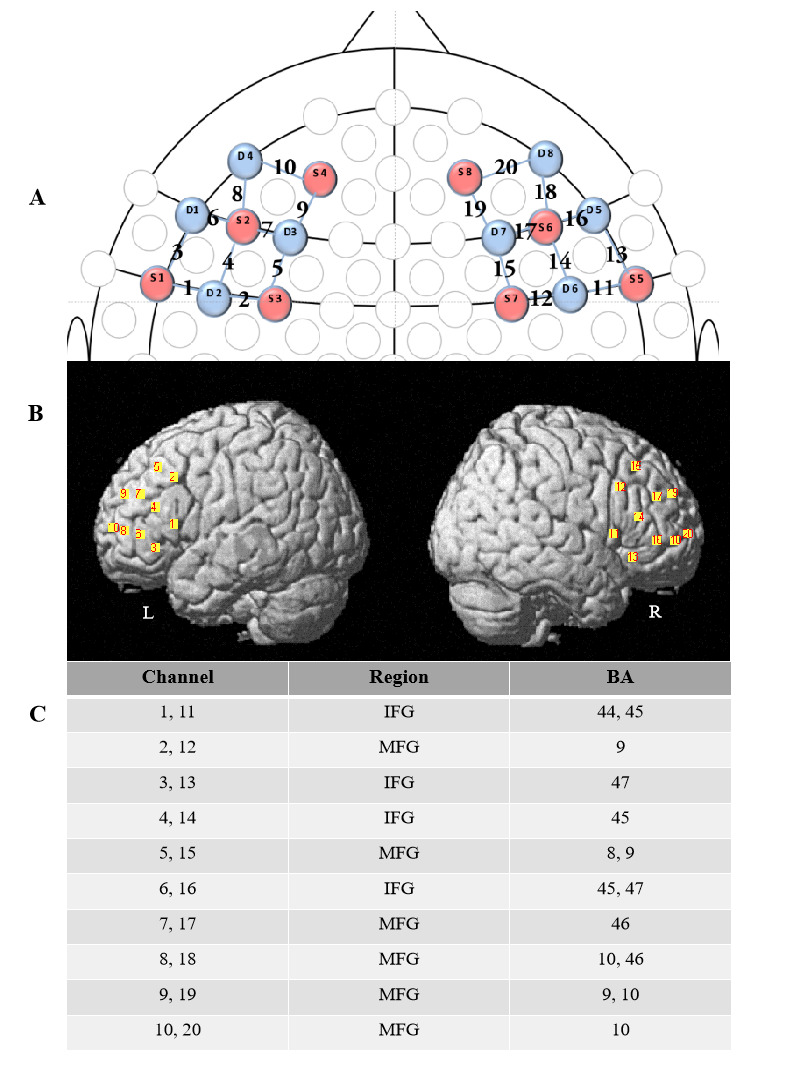
fNIRS channel and probe configuration. (A) 2d map showing an fNIRS configuration of probes (red representing sources, blue representing detectors) and channels. (B) The bilateral prefrontal cortex is overlaid by 20 channels. (C) Channels and corresponding brain regions. L: the left brain. R: the right brain. IFG: Inferior Frontal Gyrus. MFG: Middle Frontal Gyrus.

We recorded oxy-hemoglobin (HbO) and deoxy-hemoglobin (HbR) concentration changes for each child using a NIRSport 8*8 (NIRx Medical Technology LLC, Glen Head, NY, USA) Optical Topography System when the child was playing the joint attention game. Eight light sources and eight detectors were evenly distributed over the left and right prefrontal region to access brain activity. The channel montage customized for this study included 20 channels that covering bilateral DLPFC, IFG, and Middle Frontal Gyrus (MFG) (see Fig 2). Per the 10–10 transcranial positioning system, the four reference detectors (1, 3, 7, and 5) were positioned at F7, F3, F4, and F8 with equal distances of 3cm between the optodes. The absorption of near-infrared light was measured at the wavelengths of 630 and 850mm, the sampling frequency was 7.81Hz.

**Fig 3:**
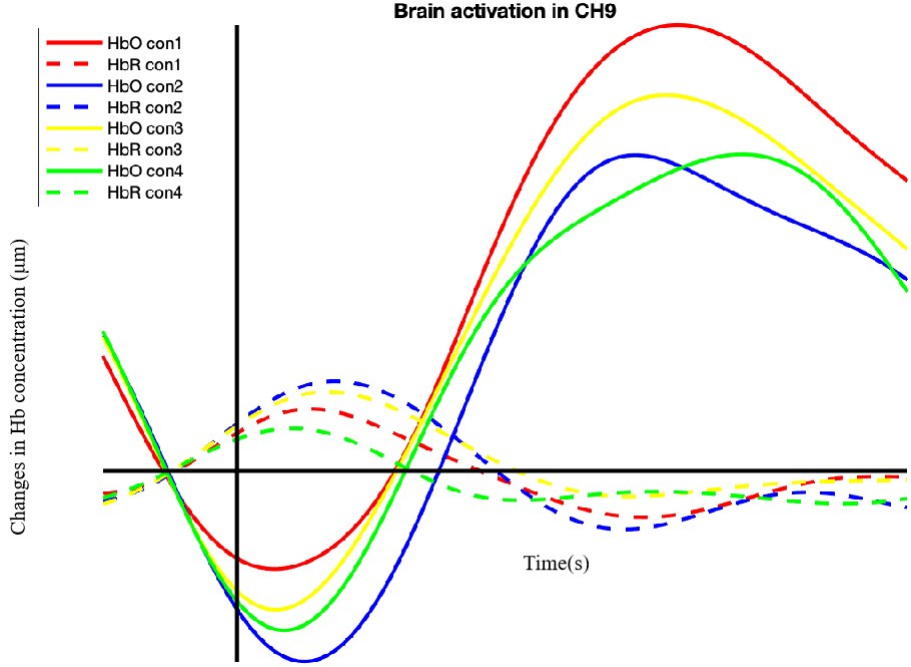
An example of grand average waveforms. Hb = Hemoglobin. HbO  =  Oxygenated hemoglobin. HbR  =  Deoxygenated hemoglobin. CH = Channel.

The fNIRS data were pre-processed using MATLAB-based (The MathWorks, Inc., Natick, MA, USA) functions derived from the toolbox Homer2 for fNIRS [28]. First, the raw intensity data were converted into optical density units before undergoing quality detection steps using the channel artifact detection method (hmrMotionArtifactByChannel) and the spline correction method (hmrMotionCorrectSpline) [29]. If any signal changes showed greater than the threshold of the standard deviation of 50 or amplifier class of 5 within 0.5s, then marked for an additional second, these artificial signal changes were corrected with a spline interpolation set to the 0.99 parameters. After that, a bandpass filter with a cutoff frequency from 0.01Hz to 0.2 Hz was employed to remove the high- and low-frequency noise. Finally, the filtered data were calculated for hemoglobin concentration changes, per the modified Beer-Lambert Law [30]. HbO concentration was selected in the following statistical analyses through Matlab coding as they were reported to be more sensitive to changes in the regional cerebral blood flow [31]. Times of interest were selected based upon visual inspection of grand-averaged activation patterns across conditions to capture peak activation for each array (see an example in Fig 3) [32]. We sought to identify the classic hemodynamic response showing an increase in HbO and a corresponding decrease in HbR when selecting a period of interest. Accordingly, 11-21s post-stimulus presentation onset was selected for the next analysis.

### Statistical analysis

Statistical analysis was conducted with IBM SPSS Statistics (IBM Corporation, Armonk, NY, USA). Paired-samples T-tests between baseline and tasks were used to investigate if there was activation during the joint attention task. We conducted a series of 2 (IJA, RJA) *2 (Stranger, teacher) repeated measures ANOVA models in each channel to examine the effect of the joint attention partners and types, and then compared both the behavioral data and brain activation of the IJA condition among three groups of preschoolers using one-way ANOVA with homogeneity of variance test first, and then using independent sample T-test in a pairwise comparison. We also assessed the correlations using bivariate Pearson’s correlations between HbO concentrations and behavioral data.

## Results

### Behavioral performance

**Fig 4:**
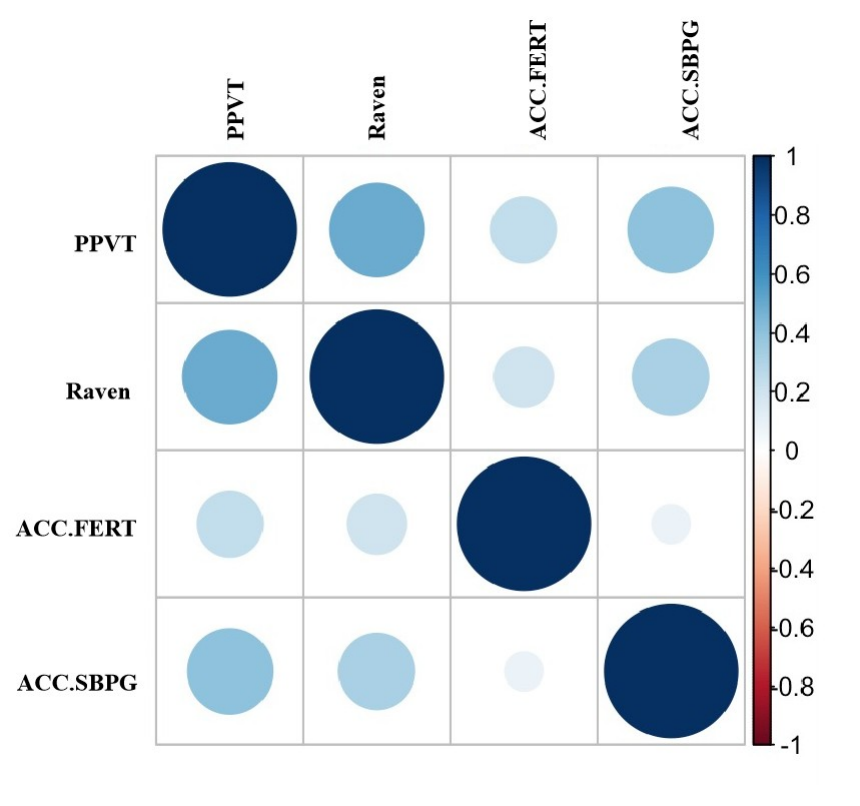
Correlations between behavior performances. Deeper color and bigger round indicate a stronger correlation. ACC.SBPG = Accuracy of Small or Big Pear game; ACC.SFERT = Accuracy of facial expression recognition task.

Table 1 presents the demographic characteristics and differences in behavioral performance among three groups of left-behind preschoolers. First, statistical differences were not detected based on preschooler’s age and the Raven Test score in three groups. Second, we found partially-left-behind children had significantly higher scores in the PPVT than completely-left-behind children (*t* = 2.415, *p* = 0.02, Cohen’s D = 0.73) and non-left-behind children (*t* = 2.378, *p* = 0.021, Cohen’s D = 0.65). Third, partially-left-behind children had a higher family monthly income than non-left-behind children (*t* = 2.278, *p* = 0.028, Cohen’s D = 0.77). Furthermore, there was no statistical difference between the accuracy of SBPG (a cognitive flexibility test) and FERT (a facial expression recognition test). Last, we found that the PPVT was associated with the accuracy of SBPG (*r* = 0.243, *p* = 0.029) and FERT (*r* = 0.410, *p* < 0.001), respectively (see Fig 4).

**Table 1:** Demographic characteristics and behavior performances of left-behind children.

	CLB children	PLB children	NLB children
	(*N* = 26)	(*N* = 19)	(*N* = 36)
Age (means ± SD*)*	64.65 ± 7.07	65.53 ± 7.15	64.92 ± 6.32
Raven	17 ± 4.08	18.74 ± 3.92	17.44 ± 4.21
PPVT	43.15 ± 17.34	56.11 ± 18.34	45.36 ± 14.54
Income	2.96 ± 1.24	3.67 ± 0.976	2.74 ± 1.413
Accuracy of SBPG	0.73 ± 0.18	0.83 ± 0.17	0.76 ± 0.18
Accuracy of FERT	0.58 ± 0.2	0.62 ± 0.22	0.62 ± 0.21

CLB = Completely-left-behind; PLB = Partially-left-behind; NLB = Non-left-behind; SBPG = Small or Big Pear game; FERT = facial expression recognition task.

### Prefrontal activation in joint attention task

**Fig 5:**
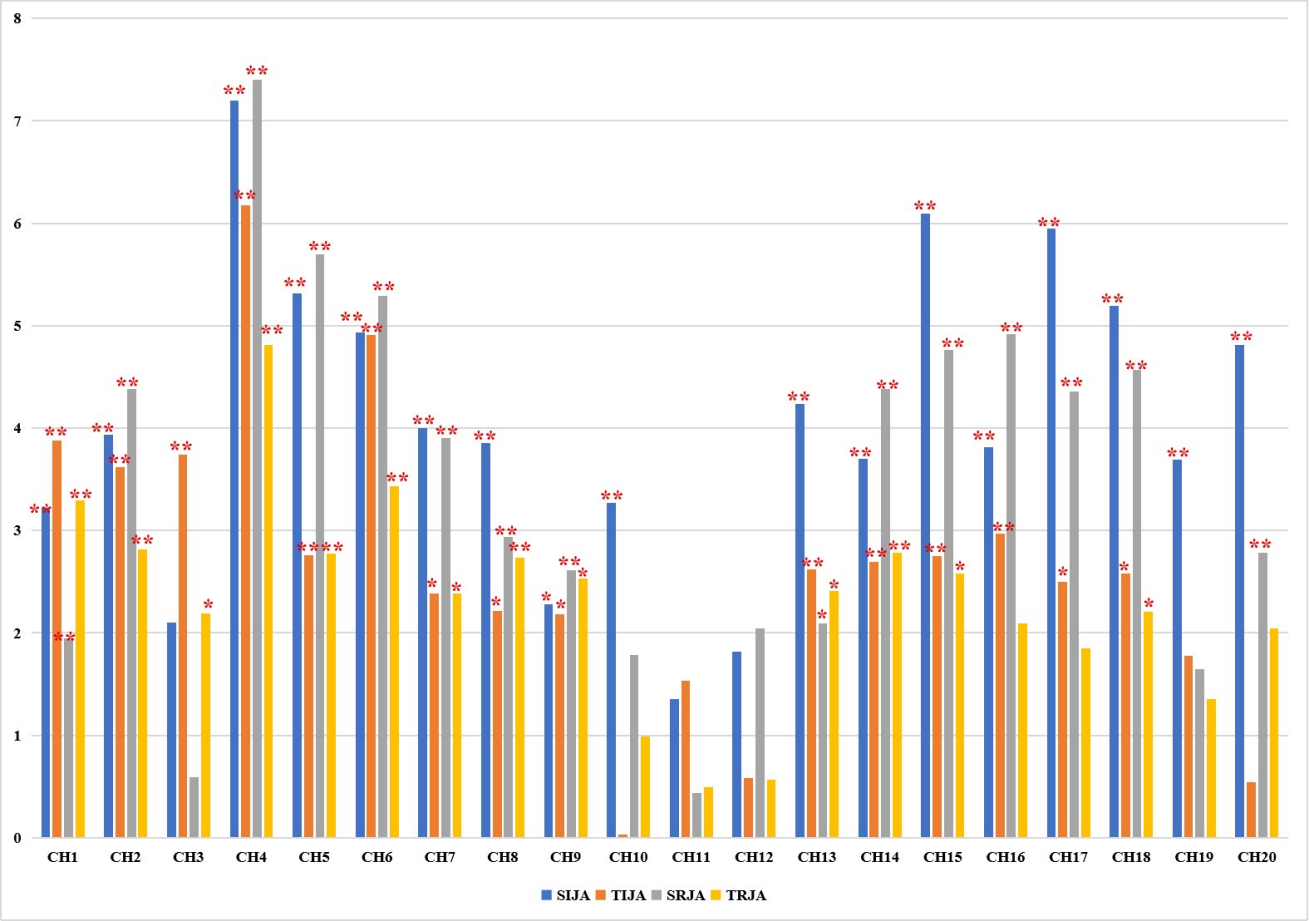
Brain activation differences between baseline and four conditions of joint attention. Bar charts show the difference of brain activation between baseline under (A) SIJA/TIJA condition, and (B) SRJA/TRJA condition, respectively. SIJA = stranger initiating joint attention; TIJA = teacher initiating joint attention; SRJA = stranger respond to joint attention; TRJA = teacher respond to joint attention. *means *p < 0*.*05*; **means *p < 0*.*01*.

Fig 5 shows the comparison of brain activity between baseline and four conditions of the joint attention task. Compared to baseline, there was significantly higher brain activation in the stranger IJA condition in CH1-CH10 and CH13-CH20 (2.1 < *t’s* < 7.19, *p’s* < 0.05), including the prefrontal area covering bilateral DLPFC and IFG. In the teacher IJA condition, CH1-CH9, CH13-CH18, and CH20 (2.18 < *t’s* < 6.18, *p’s* < 0.05) evoked higher brain activation in the bilateral prefrontal area. Moreover, children in stranger RJA condition were yielded a significantly higher brain activation than baseline in CH2, CH4-CH9, CH14-18, and CH20 (2.04 < *t’s* < 7.4, *p’s* < 0.05) in the prefrontal cortex, whereas CH1-CH9, CH13-CH15, and CH18 (2.04 < *t’s* < 4.81, *p’s* < 0.05) in the teacher RJA condition, which covered bilateral IFG and MFG (See S1 Table in S1 File for more details).

**Fig 6:**
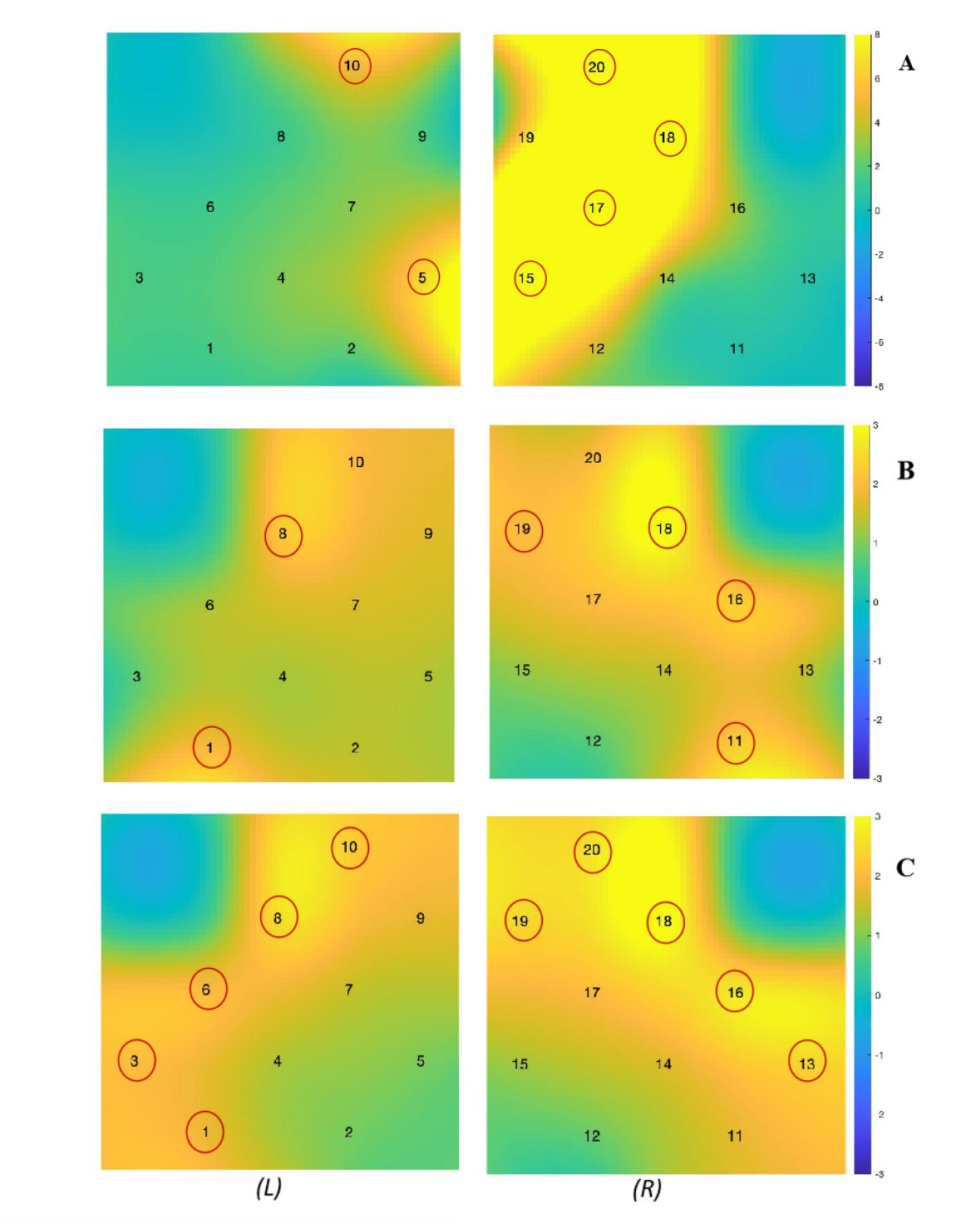
Brain activation differences heatmaps. (A) shows the main effect of results of brain activation relative to joint attention partners (i.e., stranger and teacher), in which colored bars indicate F-values. (B) and (C) display the difference of brain activation in the case of IJA condition between PLB and CLB children, and that between PLB and NLB children, respectively, in which colored bars represent the T-values. L: the left hemisphere. R: the right hemisphere.

ANOVA analysis for the 2*2 conditions (IJA/RJA * familiar/unfamiliar), revealed the significant differences in brain activation between joint attention partners (see Fig 6(A)). The result revealed a main effect of JA types in CH3 (*F* = 6.8, *p* = 0.011). Moreover, the main effect of JA partners found in CH5(*F* = 9.454, *p* = 0.0012), CH10 (*F* = 7.374, *p* = 0.027), CH15(*F* = 11.383, *p* = 0.005,), CH17(*F* = 19.722, *p* < 0.001), CH18(*F* = 10.933, *p* = 0.005), and CH20(*F* = 11.794, *p* = 0.005), indicating children evoked higher brain activation in bilateral MFG area when interacting with stranger. False discovery rate (FDR) correction was applied in all channels of brain activation [33]. Specifically, compared to teacher IJA condition, children revealed higher brain activation in stranger IJA condition in CH5 (*t* = 3.401, *p* = 0.005), CH10 (*t* = 3.364, *p* = 0.005), CH15 (*t* = 3.621, *p* = 0.003), CH17 (*t* = 3.835, *p* = 0.002), CH18 (*t* = 2.639, *p* = 0.033), and CH20 (*t* = 4.598, *p* = 0.0003) after controlling for multiple comparisons (FDR) across 20 channels [33]. (See S2 Table in S1 File for more statistics).

The ability and motivation to share attention is a unique aspect of human cognition [15] and the result of the stranger IJA and teacher IJA demonstrated the significant higher brain activation. Thus, we compared these two conditions to assess the differences among the three groups. Results showed partially-left-behind children evoked significantly higher activation in the stranger IJA condition than completely left-behind children in CH1 (*t* = 2.605, *p* = 0.013, Cohen’s D = 0.75), CH8 (*t* = 2.312, *p* = 0.028, Cohen’s D = 0.72), CH11 (*t* = 2.896, *p* = 0.006, Cohen’s D = 0.89), CH16 (*t* = 2.235, *p* = 0.031, Cohen’s D = 0.68), CH18 (*t* = 3.173, *p* = 0.003, Cohen’s D = 0.94), and CH19 (*t* = 2.121, *p* = 0.04, Cohen’s D = 0.65) (see Fig 6(B)). Similarly, in the stranger IJA condition, partially-left-behind children inspired significantly higher activation than non-left-behind children in CH1 (*t* = 2.061, *p* = 0.044, Cohen’s D = 0.58), CH3 (*t* = 2.058, *p* = 0.045, Cohen’s D = 0.64), CH6 (*t* = 2.054, *p* = 0.045, Cohen’s D = 0.59), CH8 (*t* = 2.653, *p* = 0.013, Cohen’s D = 0.78), CH10 (*t* = 2.195, *p* = 0.033, Cohen’s D = 0.61), CH13 (*t* = 2.305, *p* = 0.025, Cohen’s D = 0.61), CH16 (*t* = 2.768, *p* = 0.008, Cohen’s D = 0.79), CH18 (*t* = 3.209, *p* = 0.002, Cohen’s D = 0.9), CH19 (*t* = 2.637, *p* = 0.011, Cohen’s D = 0.73), and CH20 (*t* = 2.4, *p* = 0.02, Cohen’s D = 0.66) (see Fig 6(C)). There was no significant difference found among three groups preschooler under teacher IJA condition (See S3 Table in S1 File for more details).

### Correlations between activation and behavior data

We tested associations between preschooler’s brain activation in the joint attention tasks and behavioral performance. Firstly, we found that the PPVT was positively associated with brain activation in the stranger IJA condition in CH4 (*r* = 0.291, *p* = 0.008), CH15 (*r* = 0.288, *p* = 0.01), CH19 (*r* = 0.239, *p* = 0.033), and CH13 (*r* = 0.228, *p* = 0.042) in the teacher RJA condition. Secondly, the accuracy of FERT was positively correlated with brain activation in the stranger IJA condition in CH4 (*r* = 0.23, *p* = 0.039). Thirdly, associations between the accuracy of SBPG and brain activation were found in CH19 (*r* = 0.254, *p* = 0.024) in the stranger IJA condition and CH19 (*r* = 0.222, *p* = 0.048) in the teacher RJA condition.

## Discussion

The present study sought to evaluate the differences between two types of joint attention at the neural level and investigate the neural and behavioral differences of social cognition (i.e., joint attention, language, cognitive flexibility, and facial expression recognition) among three groups of left-behind children. First, we found that preschoolers in both the IJA and the RJA conditions yielded a higher activation than baseline in almost all prefrontal cortex including IFG, MFG, and DLPFC. Prior imaging studies demonstrated that joint attention interactions may evoke brain activation in the prefrontal and frontotemporoparietal area [17, 34, 35]. Our research focused on the prefrontal cortex and showed that preschoolers had significant activation in the prefrontal area during the joint attention task. This is consistent with the results of previous interactive joint attention studies. What is more, in early development, IJA was related to frontal cortex activity [19, 36], while RJA and its related behaviors were associated with prefrontal and temporal cortical processes [12]. This is also supported by fMRI studies on infants and adults [17, 18], which indicated that both RJA and IJA activated broad frontal networks.

Second, we found significant brain activation differences between two social interaction partners in the joint attention task that covered the bilateral prefrontal cortex including bilateral DLPFC and right MFG. The activation of the brain areas was in line with previous research [17]. This finding, however, was inconsistent with previous research; it has been reported that joint attention with a familiar person had higher activation than an unfamiliar person in children aged 8–12 years and adolescence aged 13–18 years [27]. Our finding, however, showed higher activation in the stranger IJA condition than the teacher IJA condition. The face recognition model could help to give a reasonable explanation for our finding. During joint attention, the first process is face recognition and recognition of a familiar person is a fundamental biological function for human beings [37]. The curiosity of the participant evoked by a novel stranger’s face, which could thus evoke more brain activation. Research on recognition of familiar and unfamiliar people [38] found that the connection density of the unfamiliar network was higher than that of the familiar network in theta and beta bands in an EEG study. Such a result indicates that recognition of a stranger’s face leads to a higher brain network efficiency. In addition, the researchers suggested that viewpoint-invariant responses might be found when showing faces that are familiar to the observer [38].

Based on previous findings regarding the neural responses of joint attention in children and adults, differences in prefrontal cortex activation were expected, as Schilbach et al. (2009) stated that IJA developed higher activation than RJA [39]. Our findings indicated the IJA evoked a higher activation than RJA, but no significant difference remained after controlling for the false discovery rate [33]. Prior research on joint attention found that there is a shared brain basis for IJA and RJA as shown in the prefrontal cortex. Joint attention research in adults showed in activation in the posterior superior temporal sulcus (STS), the temporoparietal junction (TPJ), and the ventral striatum [27], these areas could not be detected using this portable fNIRS device. Concerning the distinctions between the IJA and RJA conditions, it is essential to improve the current paradigm, so that participants voluntarily initiate joint attention in a naturalistic interactive task rather than being cued as in the present study.

Third, our result revealed that language level correlates with joint attention in IFG and MFG. Previous studies have indicated that joint attention provides a foundation for early language, social competence and facilitates social learning [12]. For example, Tomasello and Farrar (1986) found that the amount of time during which children and caregivers engaged in joint attention behaviors was positively correlated to children’s vocabulary development [40]. During the process of joint attention, executive function, which includes cognitive flexibility, attention, and facial expression recognition, provides critical support. Hecke et al. (2012) illustrated a better understanding of the roles of executive function in joint attention interaction, demonstrating that executive function integrated into social attention coordination [41]. Our finding found a positive correlation between joint attention and cognitive flexibility in the right PFC, which is consistent with a previous study that indicated cognitive flexibility is driven by neurodevelopmental changes in the PFC [42]. Furthermore, the association between executive parameters and joint attention may contribute to the comprehensive development of social cognition. Previous research suggested that the development of joint attention should be thought of in terms of two phases: the “learning to” and “learning from” phases [43, 44]. Children first integrate basic information processing abilities to “learn to” engage in joint attention behaviors. This “learning to” state increases the efficiency in the execution of joint attention behaviors in social interactions, and decreases the cognitive resources children must allocate to execute and manage the motor, spatial, attentional, and representational/memory skills that are supposed to be integral to learning to engage in joint attention. Therefore, successful negotiation of the “learning to” state frees sufficient cognitive and executive resources to enable children to enter the “learning from” phase of joint attention development. Children may develop abundant cognitive and executive resources to process information about self and others in this state rapidly. During this process, facial expression recognition is a significant factor in social interaction. Consequently, joint attention might develop into a type of social executive skill as it becomes more routine and efficient and provides an important platform for stimulation and the acquisition of comparative information about commonalities in perception and intentions between self and others [43].

Last but not least, we could not fully replicate previous studies’ findings that revealed deficits in left-behind preschoolers on social cognition, including language, cognitive skills, and social skills [9, 44] compared to their counterparts in urban and rural areas. In our research, partially-left-behind children presented a significantly higher level in the PPVT than completely/non-left-behind children respectively. The neural result also indicated partially-left-behind children evoked significantly higher activation in the stranger IJA condition in the IFG and MFG than completely left-behind children and non-left-behind children. Our finding thus was inconsistent with some prior behavioral research. We found no significant difference in partially-left-behind children’s intelligence (Raven test) compared to non-left-behind children, but family monthly income in partially-left-behind children was higher than non-left-behind children. The increase of family income, one benefit of parents leaving for work in an urban area, would assist children’s development in the case of children with one parent providing care. Recent research verified this conjecture, partially-left-behind children, whose one parent leaves to increase family income, and one parent stays, showed a higher level in executive function, receptive vocabulary, and social skills than completely left-behind children [8]. Additionally, the literature of the economic and social impact of migration shows that a large fraction of migrants’ incomes is devoted to remittances, which reduces the economic vulnerability of the their families [45, 46] and offers better living and educational conditions for their children.

This study has several notable strengths. First, we conducted joint attention, language, and behavioral tasks together, and we assessed left-behind children’s social cognition at both behavioral and neural levels. Second, we recruited participants’ teachers as familiar partners in the study, to assess the social interaction familiarity in joint attention tasks among three groups of left-behind children. Third, we used tablet-based tasks tailored to young children to assess participants’ cognitive flexibility and facial expression recognition. There are also several important limitations to this work that should be carefully considered for future studies. First, fNIRS imaging, although excellent for maintaining compliance and a more naturalistic assessment environment for young children, is limited to recordings from the surface of the cortex. Future work using a whole-brain method such as fMRI could provide further important insight into joint attention skills. Second, the sample diversity is relatively limited to detect the effects of all left-behind children. We only recruited in one area of rural China, which only represents a small village in one province. Future work must aim to replicate these findings in larger samples in various rural areas. Lastly, the joint attention task is quite limited in that the “interacting” partner is a static picture displayed on a screen; static pictures are relatively mechanized, which is less realistic than a live interaction. Further work should include a naturalistic task with an interactive person to evaluate the joint attention skills with familiar and unfamiliar individuals in different groups of left-behind children.

## Conclusion

In conclusion, extending previous interactive studies of joint attention, we developed a modified joint attention paradigm to explore joint attention skills with different interaction partners. This is the first study to use neuroimaging to assess children’s social-cognitive development and evaluate the differences in three groups of left-behind children, providing biological evidence of vulnerability in children’s social cognitive development. Using fNIRS to assess neural activity may offer a better understanding of social cognition in the left-behind children and social cognitive development of those children in rural China. The results also suggest that if one parent stays to care for children when the other parent leaves for work might be a better way to balance parent-child separation in rural China.

## Supporting information

S1 File (DOCX)

S1 Data (SAV)

S1 Graphical abstract (DOCX)
