# Supplementary material for: Is left-behind a real reason for children’s social cognition deficit? An fNIRS study on the effect of social interaction on left-behind preschooler’s prefrontal activation
Source: PLoS One. 2021 Sep 17;16(9):e0254010. doi: 10.1371/journal.pone.0254010 (PMC8448372; doi:10.1371/journal.pone.0254010)
Supplement: S1 File — (DOCX) [file pone.0254010.s001.docx]

# Supporting information

**S1 Fig. The signed consent form from the child’s mother, whose child’s photograph was allowed to use in publication.**


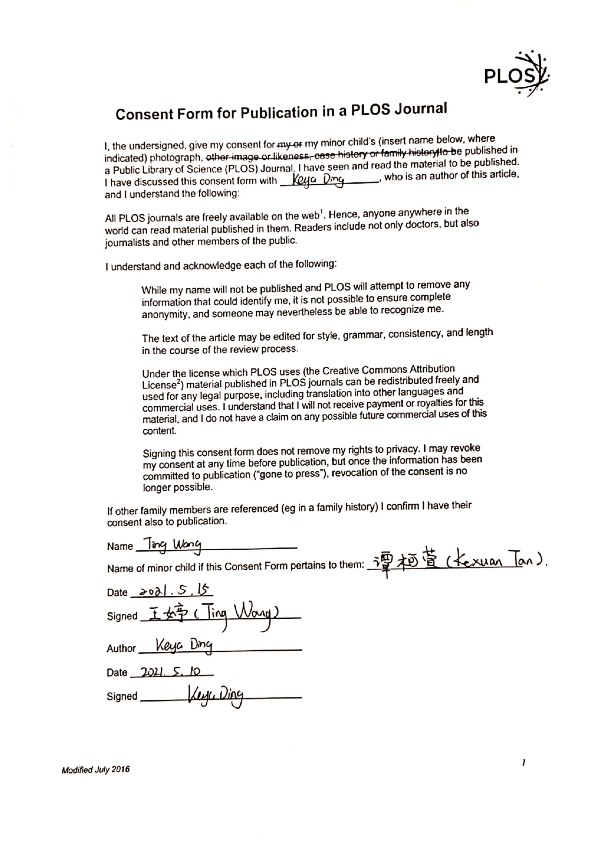


**S1 Table. Results of T-test of baseline and four conditions of the joint attention task.** SIJA= stranger-initiating joint attention; TIJA= teacher-initiating joint attention; SRJA= stranger-respond to joint attention; TRJA= teacher-respond to joint attention.

| Channel | Baseline vs. SIJA | | Baseline vs. TIJA | | Baseline vs. SRJA | | Baseline vs. TRJA | |
| --- | --- | --- | --- | --- | --- | --- | --- | --- |
|  | *t* | *p* | *t* | *p* | *t* | *p* | *t* | *p* |
| 1 | 3.22171987 | 0.00186824 | 3.87774094 | 0.00022048 | 1.94227622 | 0.05576054 | 3.29275178 | 0.00149985 |
| 2 | 3.93392788 | 0.00017849 | 3.61378991 | 0.00052896 | 4.38391475 | 3.55E-05 | 2.81671547 | 0.00612557 |
| 3 | 2.09616246 | 0.0393527 | 3.74175468 | 0.00035003 | 0.59130461 | 0.55604828 | 2.19020568 | 0.03153608 |
| 4 | 7.19481718 | 2.99E-10 | 6.17808584 | 2.56E-08 | 7.40037908 | 1.20E-10 | 4.81184931 | 6.93E-06 |
| 5 | 5.31743511 | 9.53E-07 | 2.75859509 | 0.00721036 | 5.69454464 | 2.03E-07 | 2.77717702 | 0.00684578 |
| 6 | 4.93395703 | 4.55E-06 | 4.91146492 | 4.96E-06 | 5.29296244 | 1.10E-06 | 3.43013456 | 0.00097256 |
| 7 | 3.99685956 | 0.000142 | 2.38461205 | 0.01946393 | 3.89798418 | 0.00020054 | 2.38141327 | 0.01962218 |
| 8 | 3.85004263 | 0.00023664 | 2.21250352 | 0.02978269 | 2.93693008 | 0.0043278 | 2.73442066 | 0.00769211 |
| 9 | 2.27581002 | 0.02553178 | 2.18296466 | 0.03197003 | 2.61185567 | 0.01075128 | 2.52652464 | 0.01349201 |
| 10 | 3.27173608 | 0.00157882 | 0.02830296 | 0.97749101 | 1.78094785 | 0.0787169 | 0.98874912 | 0.32576703 |
| 11 | 1.35116935 | 0.18049814 | 1.53385293 | 0.1290611 | 0.43593222 | 0.66407483 | 0.49287395 | 0.62346884 |
| 12 | 1.8172045 | 0.0729778 | 0.57888063 | 0.56431661 | 2.04135551 | 0.04455418 | 0.5625635 | 0.57532511 |
| 13 | 4.23493686 | 6.20E-05 | 2.61734533 | 0.01063962 | 2.09047482 | 0.03983244 | 2.40960478 | 0.01832632 |
| 14 | 3.69812835 | 0.00039942 | 2.69395886 | 0.00862111 | 4.37945282 | 3.61E-05 | 2.7826079 | 0.00674246 |
| 15 | 6.09679388 | 4.02E-08 | 2.74869275 | 0.00745075 | 4.7625383 | 8.81E-06 | 2.5764373 | 0.01189518 |
| 16 | 3.81445447 | 0.00026738 | 2.97071484 | 0.00392163 | 4.91827344 | 4.58E-06 | 2.09570396 | 0.03927034 |
| 17 | 5.94683488 | 6.84E-08 | 2.4946432 | 0.01466771 | 4.35560835 | 3.90E-05 | 1.84659146 | 0.06850439 |
| 18 | 5.19555713 | 1.56E-06 | 2.57955576 | 0.01174711 | 4.56543328 | 1.81E-05 | 2.2063599 | 0.03026327 |
| 19 | 3.69418975 | 0.00040473 | 1.77450159 | 0.07983274 | 1.64872058 | 0.10317635 | 1.35116141 | 0.18050068 |
| 20 | 4.81363312 | 6.89E-06 | 0.54524953 | 0.5870989 | 2.78128337 | 0.00674976 | 2.04219833 | 0.0444265 |

**S2 Table. Results of the two-factor repeated measures ANOVA in the joint attention task.** JA= joint attention.

|  | Main effect of JA types | | Main effect of JA objects | | Interaction effect | |
| --- | --- | --- | --- | --- | --- | --- |
| Channel | *p* | *F* | *p* | *F* | *p* | *F* |
| 1 | 0.074 | 3.278 | 0.286 | 1.154 | 0.677 | 0.174 |
| 2 | 0.998 | 0 | 0.38 | 0.779 | 0.461 | 0.549 |
| 3 | 0.011 | 6.8 | 0.186 | 1.778 | 0.989 | 0 |
| 4 | 0.467 | 0.533 | 0.103 | 2.716 | 0.51 | 0.438 |
| 5 | 0.961 | 0.002 | 0.003 | 9.454 | 0.236 | 1.429 |
| 6 | 0.282 | 1.172 | 0.253 | 1.328 | 0.529 | 0.4 |
| 7 | 0.784 | 0.076 | 0.084 | 3.058 | 0.439 | 0.605 |
| 8 | 0.992 | 0 | 0.228 | 1.473 | 0.165 | 1.96 |
| 9 | 0.894 | 0.018 | 0.913 | 0.012 | 0.569 | 0.326 |
| 10 | 0.686 | 0.165 | 0.008 | 7.374 | 0.055 | 3.791 |
| 11 | 0.102 | 2.731 | 0.766 | 0.089 | 0.996 | 0 |
| 12 | 0.859 | 0.032 | 0.085 | 3.049 | 0.957 | 0.003 |
| 13 | 0.07 | 3.366 | 0.964 | 0.002 | 0.405 | 0.701 |
| 14 | 0.572 | 0.322 | 0.175 | 1.871 | 0.998 | 0 |
| 15 | 0.273 | 1.221 | 0.001 | 11.383 | 0.18 | 1.834 |
| 16 | 0.784 | 0.075 | 0.14 | 2.223 | 0.372 | 0.806 |
| 17 | 0.098 | 2.801 | 0 | 19.722 | 0.211 | 1.593 |
| 18 | 0.526 | 0.406 | 0.001 | 10.933 | 0.448 | 0.582 |
| 19 | 0.153 | 2.081 | 0.234 | 1.437 | 0.143 | 2.189 |
| 20 | 0.787 | 0.074 | 0.001 | 11.794 | 0.005 | 8.359 |

**S3 Table. Results of brain activation differences among three groups of left-behind children.** CLBC=completely-left-behind children; PLBC=partially-left-behind children; NLBC= Non-left-behind children.

| Channel | C-LBC vs. P-LBC | | C-LBC vs. N-LBC | | P-LBC vs. N-LBC | |
| --- | --- | --- | --- | --- | --- | --- |
|  | *t* | *p* | *t* | *p* | *t* | *p* |
| 1 | -2.605 | 0.013 | -0.273 | 0.786 | 2.061 | 0.044 |
| 2 | -1.475 | 0.148 | -0.851 | 0.398 | 0.79 | 0.433 |
| 3 | -0.327 | 0.745 | 2.12 | 0.038 | 2.058 | 0.045 |
| 4 | -1.261 | 0.214 | -0.083 | 0.934 | 1.237 | 0.222 |
| 5 | -1.19 | 0.241 | -0.571 | 0.57 | 0.795 | 0.43 |
| 6 | -1.253 | 0.217 | 1.318 | 0.193 | 2.054 | 0.045 |
| 7 | -1.61 | 0.115 | -0.567 | 0.573 | 1.41 | 0.165 |
| 8 | -2.313 | 0.028 | 0.59 | 0.558 | 2.653 | 0.013 |
| 9 | -1.684 | 0.099 | -0.099 | 0.921 | 1.856 | 0.069 |
| 10 | -1.916 | 0.062 | 0.458 | 0.648 | 2.195 | 0.033 |
| 11 | -2.896 | 0.006 | -1.476 | 0.145 | 1.767 | 0.083 |
| 12 | -0.354 | 0.725 | 0.049 | 0.961 | 0.448 | 0.656 |
| 13 | -0.814 | 0.42 | 1.511 | 0.136 | 2.305 | 0.025 |
| 14 | -1.397 | 0.17 | -0.186 | 0.853 | 1.566 | 0.123 |
| 15 | -0.91 | 0.368 | -0.108 | 0.914 | 0.995 | 0.324 |
| 16 | -2.235 | 0.031 | 0.413 | 0.681 | 2.768 | 0.008 |
| 17 | -1.974 | 0.055 | -0.215 | 0.831 | 1.973 | 0.054 |
| 18 | -3.173 | 0.003 | 0.024 | 0.981 | 3.209 | 0.002 |
| 19 | -2.121 | 0.04 | 0.094 | 0.925 | 2.637 | 0.011 |
| 20 | -1.56 | 0.126 | 0.899 | 0.372 | 2.4 | 0.02 |
