# Supplementary figures and images for: Is left-behind a real reason for children’s social cognition deficit? An fNIRS study on the effect of social interaction on left-behind preschooler’s prefrontal activation
Source: PLoS One. 2021 Sep 17;16(9):e0254010. doi: 10.1371/journal.pone.0254010 (PMC8448372; doi:10.1371/journal.pone.0254010)

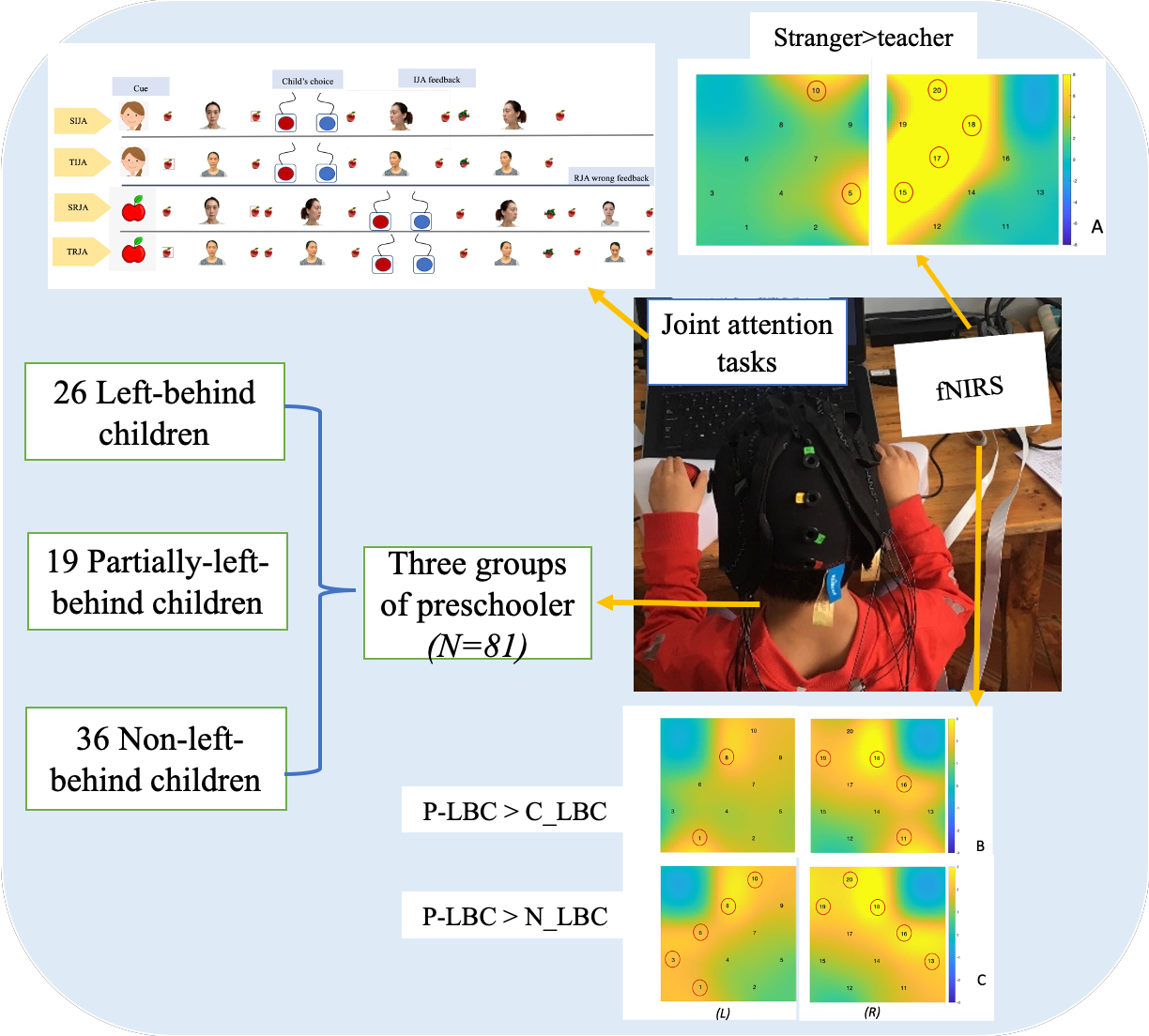

Supplement: S1 Graphical abstract — (DOCX) [file pone.0254010.s003.docx]
